# The gut–brain axis in Alzheimer’s disease is shaped by commensal gut microbiota derived extracellular vesicles

**DOI:** 10.1080/19490976.2025.2501193

**Published:** 2025-05-12

**Authors:** Junhua Xie, Lien Van Hoecke, Elien Van Wonterghem, Griet Van Imschoot, Vanessa Andries, Lars Vereecke, Roosmarijn E. Vandenbroucke

**Affiliations:** a VIB Center for Inflammation Research, VIB, Ghent, Belgium; b Department of Biomedical Molecular Biology, Ghent University, Ghent, Belgium; c Department of Internal Medicine and Pediatrics, Ghent University, Ghent, Belgium; d Ghent Gut Inflammation Group (GGIG), Ghent University, Ghent, Belgium

**Keywords:** Gut microbiota, bacterial extracellular vesicles, Alzheimer’s disease, gut–brain axis

## Abstract

Emerging clinical and experimental evidence highlight the involvement of gut microbiota in the onset and progression of neurodegenerative diseases such as Alzheimer’s disease (AD) via neuroinflammatory processes along the gut–brain axis. Despite this, the precise mechanisms governing gut microbial involvement in AD remain elusive. In this study, we observed that *App*^*NL-G-F*^ AD mice raised under germ-free (GF) conditions, display a reduced amyloid-β (Aβ) pathology, accompanied by a shift in microglial cells toward a less inflammatory state and increased phagocytotic efficiency. In addition, we demonstrate that gut microbiota depletion can protect against synaptic deficits in AD mice. Notably, administering bacterial extracellular vesicles (bEVs), i.e. nano-sized particles packed with bacterial components, derived from fecal slurry from specific pathogen-free housed *App*^*NL-G-F*^ AD mice, reversed the effects of GF conditions on both microglial activation and Aβ plaque accumulation. These findings reveal for the first time that commensal gut microbiota-derived bEVs have a major impact on AD pathology progression.

## Introduction

Alzheimer’s disease (AD) is a progressive neurodegenerative disorder and the most common cause of dementia. Despite significant research efforts, we are currently unable to effectively address the cognitive decline associated with Alzheimer’s, which is projected to affect a staggering 150 million people by 2050.^1,2^ For decades, research has mainly focused on the deposition of amyloid-beta (Aβ) protein in senile plaques outside the neurons and the formation of neurofibrillary tangles (NFT) composed of hyperphosphorylated Tau (p-Tau) protein inside the neurons. These processes lead to the loss of synapses, ultimately resulting in neurodegeneration and cognitive decline in AD.^3^ During the last years, it became increasingly clear that both peripheral and central inflammation contribute to AD pathogenesis, often as an early process, and potentially links Aβ amyloidosis with neurodegeneration.^4–6^ Recent FDA-approved anti-amyloid monoclonal antibodies, Aducanumab and Lecanemab, reduce amyloid burden on PET scans but lack significant improvement in Alzheimer’s patients’ cognitive performance or quality of life.^1,2^ This underscores our current inability to effectively meet patients’ urgent need for an improved quality of life, highlighting the necessity of exploring alternative approaches capable of effectively interfering with the disease’s progression.

One such alternative approach gaining increasing attention in recent years is the involvement of the gut–brain axis. The gut microbiota comprises a diverse community of microorganisms inhabiting the gastrointestinal tract. Alterations in its composition have been linked to various gastrointestinal and metabolic disorders such as inflammatory bowel disease, obesity, diabetes, and insulin resistance.^7^ More recently, the impact of the gut microbiota on central nervous system (CNS) function has garnered significant attention and alterations in gut microbiota composition have been linked to various neurological conditions, including AD.^8^ In addition to human studies in AD patients, altered microbiomes have also been reported in various AD mouse models.^9,10^ In germ-free APP/PS1 Tg mice, reduced Aβ deposition compared with their specific pathogen-free counterparts has been observed and colonization of germ-free mice with the conventional mice’s microbiota increased Aβ pathology.^11^ Going beyond, a clinical trial (NCT03808389) investigating the safety and efficacy of fecal microbiota transplantation (FMT) from healthy donors in patients with mild-to-moderate Parkinson’s disease (PD) found that a single FMT induced mild but long-lasting beneficial effects on motor symptoms in early-stage PD patients.^12^ While the above studies suggest that patients with neurological conditions have an altered gut microbiome, and that manipulations to the gut microbiome can alter the pathology, the precise mechanisms by which the gut microbiota influences brain pathology remain to be elucidated.

Looking into the mechanism behind the gut–brain axis, bacteria populating the gut microbiota can excrete large quantities of lipopolysaccharides (LPS) and amyloids, which might contribute to the modulation of signaling pathways and the production of proinflammatory cytokines associated with the pathogenesis of AD.^6,13,14^ Moreover, the gut microbiota and its metabolites, such as short-chain fatty acids (SCFAs), can influence the maturation and function of microglia in the CNS and the sealing capacity of brain barriers, which are both important for brain homeostasis.^15–17^ Next to these bacterial factors, growing interest surrounds bacterial extracellular vesicles (bEVs) as carriers of biological signals between bacteria and the host.^18,19^ Ranging from 20 to 400 nm in diameter, bEVs carry diverse cargo’s, including LPS, peptidoglycan, proteins, toxins, metabolites, and nucleic acids.^20,21^ Production of bEVs enables bacteria to deliver a multitude of effector molecules to distant target cells in a concentrated and protected manner, thereby regulating recipient cell function.^22^ Notably, certain gut microbes, including *Helicobacter pylori* (*H. pylori*), *Porphyromonas gingivalis* (*P. gingivalis*), and *Escherichia coli* (*E. coli*), produce EVs capable of accessing the brain via the bloodstream and the vagus nerve.^22–24^ Upon reaching the brain, these bEVs are internalized by brain cells such as neurons and astrocytes, potentially contributing to brain dysfunction and AD pathology, eventually leading to memory decline.^23,24^ Furthermore, bEVs from the fecal microbiota of AD patients have demonstrated the ability to penetrate the brain, exacerbating cognitive impairment in healthy mice.^25^ Despite these compelling findings suggesting a role for bEVs from the gut microbiota in influencing AD pathology, the underlying mechanisms remain largely unexplored.

Here, we further build on these findings and explore the effect of the commensal gut microbiota and the mechanism underlying these effects in the *App*^*NL-G-F*^ AD mice, a second-generation AD mouse model that avoids the typical overexpression artifacts of most AD mouse models. Instead, the APP gene containing three mutations associated with familial AD resulting in elevated levels of pathogenic Aβ, is knocked in under the control of the endogenous promotor.^26^ We show that the absence of the gut microbiota reduces Aβ pathology and synaptic deficits in *App*^*NL-G-F*^ AD mice, and that this is associated with increased microglial activation. Remarkably, gut microbiota-derived bEVs alone are capable of promoting Aβ pathology in *App*^*NL-G-F*^ AD mice and changes in microglial activation and phagocytosis may contribute to this effect. These findings strongly indicate that commensal gut microbiota-derived bEVs play a critical role in the pathogenesis of AD.

## Material and methods

### Animals

Wild-type C57BL/6J and *App*^*NL-G-F*^ mice (carrying Arctic, Swedish, and Beyreuther/Iberian mutations)^27^ were bred at a specific-pathogen-free (SPF) facility. Mice were kept in individually ventilated cages under a 14-h dark/10-h light cycle and received food and water *ad libitum*. GF *App*^*NL-G-F*^ mice were generated via axenic embryo transfer at the Germ-free and Gnotobiotic Mouse Facility Ghent (GFMF), in collaboration with the VIB/IRC Transgenic Core Facility. GF mice were housed and bred in ‘open’ cages in positive-pressure flexible film isolators (North Kent Plastics). They were transferred to individually ventilated positive-pressure Isocage-P cages (Tecniplast) and left to acclimatize for 1 week. Both male and female mice (4 and 12 months of age), as well as age-matched control wild-type littermates were used. Liquids and solids in the germ-free facility were sterilized as displayed in Table 1. Note that SPF mice were fed with the same autoclaved diet (Teklad 2018S) as was used for the GF mice. The animals were randomly allocated to experimental groups. To ensure the sterility of drinking water and food in the germ-free facility, the following sterilization cycles were applied for liquids and solids.

**Table 1.** Sterilization cycles for liquids and solids in the germ-free facility.

Program	Sterilisation	Drying	
Germ Free liquid	121°C, 30 min		Pressure pre-phase: 2000 mbar
			Pressure post-phase: 2000 mbar
			Initial pre-phase: 200 mbar
			initial sterilisation: 150 mbar
Germ Free solid	132°C, 20 min	60 mbar, 15 min	Pressure pre-phase: 2300 mbar
			pressure post-phase: 3000 mbar
			Initial pre-phase: 200 mbar
			Initial sterilisation: 150 mbar

All animal studies were conducted in compliance with governmental and EU guidelines for the care and use of laboratory animals and were approved by the ethical committee of the Faculty of Medicine and Health Sciences and Faculty of Sciences, Ghent University, Belgium.

### Bacterial extracellular vesicles isolation and purification

The gut microbiota-derived bEVs were isolated and purified using a combination of ultracentrifugation (UC), size exclusion chromatography (SEC), and density gradient centrifugation (DGC) as previously described.^28^ Specifically, the gut contents from the cecum and colon were collected and diluted in 10 times endotoxin-free PBS followed by rotation at 37°C for 30 min. The samples were centrifuged at 8,000 × g for 15 min at 4°C twice to remove bacterial debris and large contaminants. The supernatants were filtered sequentially using 0.45 and 0.22 μm membrane filters and concentrated 50 times using a 10 kDa cutoff centrifugal filter. The crude bEVs were subsequently isolated by DGC. A discontinuous iodixanol gradient was prepared by layering 4 ml of 50%, 4 ml of 40%, 4 ml of 20%, 3.5 ml of 10% iodixanol, and 1 ml of PBS in a 17 ml ultracentrifugation tube (Beckman Coulter). The 50% layer was obtained by mixing 667 µl of the sample with 3.33 ml Optiprep. The DG was centrifuged at 100,000 g for 18 h at 4°C (rotor SW 32.1 Ti and centrifuge Optima L-90K, Beckman Coulter). DG fractions #1–16 of 1 ml were collected, and EV-contained fractions (1.133–1.201 g/ml) by measuring the density of the fractions at OD_340 nm_ (Eppendorf BioPhotometer).^28^ Subsequently, fractions #7–11 were pooled, and the bEVs in these fractions were further purified by using commercially available qEV2/35 nm column (Izon Science) to remove the OptiPrep medium and EEVs. In SEC, the bEV-contained fractions #7–11 were pooled and concentrated again using a 10 kDa cutoff centrifugal filter. The number of bEV particles was measured by nanoparticle tracking analysis (NTA) using a Zetaview system (Particle Metrix, Germany). The yield of bEVs was ~2 × 10^11^ particles per gram of gut contents. bEV samples isolated from different batches were pooled, aliquoted, and stored in PBS at −80°C prior to characterization and functional experiments. To exclude that the observed effect was due to non-bacterial derived particles contamination, the bEV isolation and purification protocol was in parallel also applied to the gut contents from GF mice. Briefly, the same amount of gut content was used to undergo UC, SEC, and DGC separation and purification, the same fractions were collected, and concentrated to equal volumes. These samples were used as control in all our experiments.

### LAL assay

LPS activity levels of isolated bEVs were measured using the Limulus Amebocyte Lysate (LAL) assay (Associates of Cape Cod, Massachusetts, USA) according to the manufacturer’s protocol. The protein content of bEV samples was assessed by Pierce BCA protein assay kit (Thermo Scientific) according to the manufacturer’s instructions.

### Transmission electron microscopy

Purified OMVs were visualized by negative staining TEM as described previously.^23,29^ In short, samples were spotted on a parafilm sheet. Next, formvar/C-coated hexagonal copper grids (EMS G200H-Cu), which were glow discharged for 10 sec, were placed on top of the droplet for 1 min with the coated side of the grid down. The grids were washed 5 times in droplets of Milli-Q water, stained with 1% (w/v) uranyl acetate for 10 sec and air dried for 24 h before imaging. Visualization of the samples was done using a JEM 1400plus TEM (JEOL, Tokyo, Japan) operating at 80 kV.

### Bacterial extracellular vesicles treatment

bEV treatments were performed as described previously.^23^ In brief, mice were simultaneously administered intrarectally and orally with equal volumes of gut microbiota-derived bEVs (2 × 10^10^ particles per injection) or an equivalent volume of control sample alone 3 times per week (Monday, Wednesday, and Friday) for 3 weeks.

### Immunohistochemistry

Immunostainings on the mouse brain was performed as described previously.^23,29^ In short, the sections were cut depending on the processing method used, namely 5 µm for paraffin and 50 µm for vibratome sections. Paraffin sections were de-paraffinized in xylene and ethanol, boiled in citrate buffer for 20 min, and followed by blocking with 5% goat serum in PBS-T (PBS containing 0.3% Triton X-100) solution for 1 h at room temperature. The sections were then stained with primary antibodies in a blocking buffer at 4°C overnight. After washing with PBS, sections were stained with appropriate fluorophore-conjugated secondary antibodies in PBS containing 0.1% Triton X-100 for 1.5 h before washing and mounting. Vibratome sections were treated with a blocking buffer directly and followed the same steps as above for paraffin staining. The following primary antibodies were used: anti-GFAP (cat. no. Z033429–2, Agilent; 1:500); anti-IBA1 (cat. no. 019–1941, Wako; 1:500); anti-β-Amyloid (cat. no. 803001, BioLegend; 1:500); anti-NeuN (cat. no. MAB377, Merck; 1:500); anti-PSD-95 (cat. no. MA1–045, Thermo Fisher Scientific; 1:400); anti-Synaptophysin (cat. no. ab32127, Abcam; 1:200); and anti-CD68 (cat. no. ab53444, Abcam; 1:100). A Zeiss LSM780 confocal microscope or Zeiss Axioscan Z.1 was used for imaging. Images were processed using Image J, and the intensity of overlapping signals were quantified with Colocalization analysis for Image J.

### Glial cell morphology quantification

The quantification of glial cell morphology was performed as described previously.^23,29^ In short, IBA1-positive microglia and GFAP-positive astrocytes were imaged with a 463× oil objective using the confocal microscope with the z-stack model. Images were analyzed using a filament tracing algorithm from Imaris software (Bitplane).

### Synaptic imaging and quantification

Synaptic imaging and quantification were performed as described previously.^23,29^ In short, brain paraffin sections were co-immunostained with the anti-SYP and anti-PSD-95 antibodies and imaged with the 63× oil objective with the 3× zoom using confocal microscopy. Images were processed using Image J and the number of colocalized puncta was quantified with the Synapse Counter plugin for Image J.^30^

For quantification of PSD-95 and SYP inside CD68-positive phagosomes and IBA-positive microglia, brain vibratome sections were co-immunostained with anti-IBA1, anti-CD68, and anti-PSD-95 antibodies. Sections were imaged with the 63× oil objective using a confocal microscope with the z-stack model. Images were analyzed using the surface function from Imaris software.

### Statistical analysis

All data were presented as mean ± SEM. Mann–Whitney U tests were used to determine the statistical significance between two independent groups. The Kruskal–Wallis test followed by Dunn’s post hoc test was used to determine statistical significance among multiple groups. A *p* value of <0.05 was considered statistically significant.

## Results

### The absence of gut microbiota reduces the Aβ burden in App^NL-G-F^ AD mice and is associated with increased microglial localization to Aβ plaques

**Figure 1.**
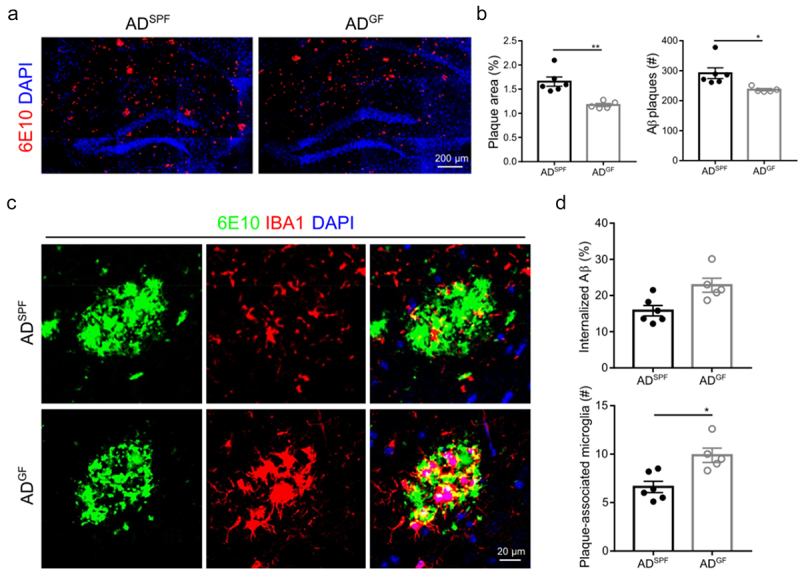
Germ-free housing reduces hippocampal Aβ deposition in *App*^*NL-G-F*^ AD mice. (a–b) Representative images (a) and of and quantification (b) of 6E10 staining in the hippocampus. (c–d) Representative images (c) and quantification of IBA1^+^ microglia and 6E10 staining in the hippocampus. 3–5 plaques (> 600 μm^2^) are analyzed per mouse. The graphs are shown as the mean ± SEM and the datapoints are biological replicates (*n* = 5–6). Statistical significance was determined by Mann–Whitney U test. *p < 0.05, **p < 0.01. SPF: specific pathogen-free; GF: germ-free; AD: *App*^*NL-G-F*^ mouse model of Alzheimer’s disease.

Comparing the Aβ plaque load between germ-free (GF) and specific pathogen-free (SPF) housing in the APP knock-in (*App*^*NL-G-F*^) mouse model shows that the area and number of Aβ plaques are diminished in the hippocampus of GF housed *App*^*NL-G-F*^ AD mice compared to SPF housed *App*^*NL-G-F*^ AD mice (Figure 1(a,b)). As microglia are one of the important players in the brain mediating Aβ clearance as well as limiting plaque growth and accumulation,^31^ we investigated the amount of internalized Aβ and Aβ plaque-associated microglia via IBA1 and 6E10 co-immunostaining. We observed an increase in number of microglia adjacent to the large plaques (>600 mm^2^) in GF housed *App*^*NL-G-F*^ AD mice compared to SPF housed *App*^*NL-G-F*^ AD controls (Figure 1(c,d)). Taken together, these findings indicate that the absence of gut microbiota reduces Aβ pathology, which is associated with an increased colocalization of microglia with Aβ plaques.

### The absence of gut microbiota affects microglia in both WT and App^NL-G-F^ AD mice, while only astrocytes are in WT mice

To determine the effects of the gut microbiota on glial cells, we investigated both WT and *App*^*NL-G-F*^ AD mice housed under SPF *versus* GF housing.

**Figure 2.**
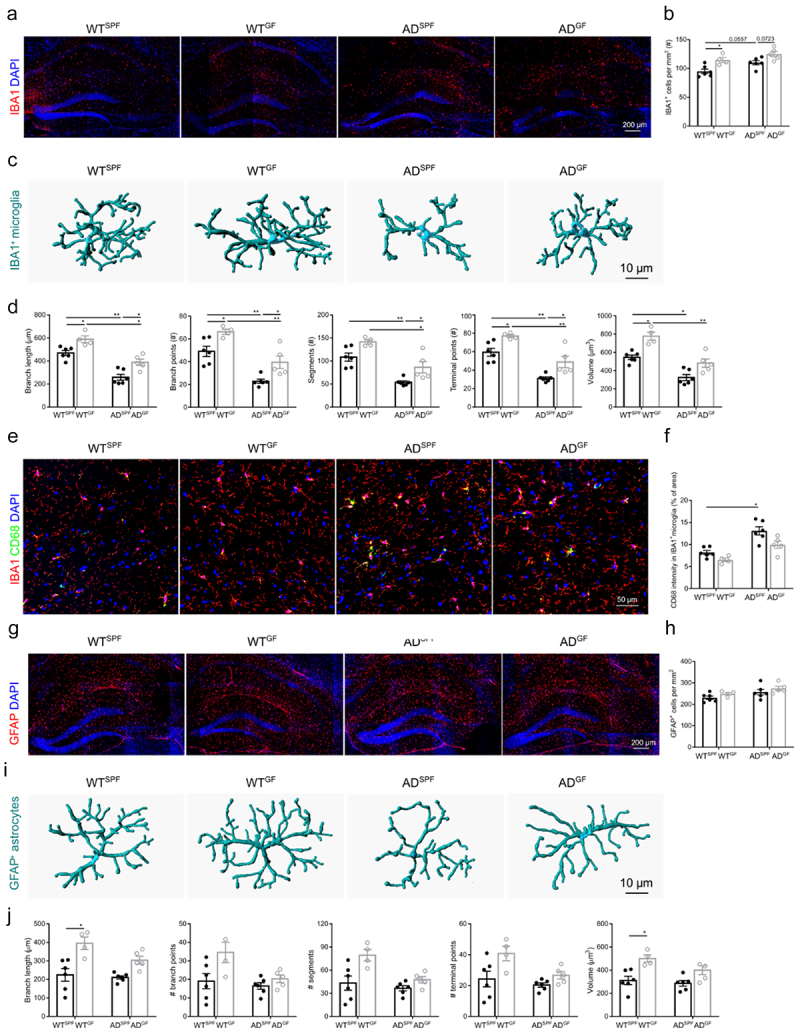
Germ-free housing affects microglial response in both WT and *App*^*NL-G-F*^ AD mice, while only astrocyte responses are affected in WT mice. (a–b) Representative images (a) and quantification of the number (b) of IBA1^+^ microglia in hippocampus. (c–d) Imaris-based 3D morphometric reconstruction analysis (c) and quantification of cell morphology (D) of IBA1^+^ microglia in hippocampus. (e–f) Representative images (e) and quantification (f) of IBA1 and CD68 staining in hippocampus. (g–h) Representative images (g) and quantification of GFAP^+^ astrocytes in hippocampus (h). (i–j) Imaris-based 3D morphometric reconstruction analysis (i) and quantification of cell morphology (j) of GFAP^+^ astrocytes in hippocampus. The graphs are shown as the mean ± SEM and the datapoints are biological replicates (*n* = 4–6). Statistical significance was determined by Kruskal–Wallis test followed by Dunn’s post hoc test comparisons. *p < 0.05, **p < 0.01. SPF: specific pathogen-free; GF: germ-free; AD: *App*^*NL-G-F*^ mouse model of Alzheimer’s disease.

Quantification of the number of IBA1^+^ microglia cells in the hippocampus revealed a significantly higher microglial density in the hippocampus of WT mice housed under GF conditions compared to SPF WT mice. A similar trend, although not significant, was observed in the *App*^*NL-G-F*^ AD mice (Figure 2(a,b)). In line with our previous findings,^6^ SPF *App*^*NL-G-F*^ AD mice contain more IBA^*+*^ microglia in the hippocampus compared to SPF WT mice.

In addition, quantitative morphometric 3D analysis of microglia revealed that the absence of gut microbiota, both in WT and *App*^*NL-G-F*^ AD mice, resulted in longer processes and increased numbers of segments, branching, terminal points, and volume of IBA1^+^ microglia, typical for a more ramified microglial morphology (Figure 2(c,d)). Also, here in line with our previous findings,^6^ SPF *App*^*NL-G-F*^ AD mice contain a more amoeba-like microglia in the hippocampus compared to SPF WT mice.

Next, we investigated the phagocytic activity of Aβ-plaque-associated microglia using IBA1 and CD68 co-immunostaining. Our results reveal that GF housing lowers CD68 levels in IBA1^+^ cells of *App*^*NL-G-F*^ AD mice (Figure 2(e,f)), with a similar trend observed in WT mice. Moreover, SPF *App*^*NL-G-F*^ AD mice display a significantly increased CD68 expression in microglia compared to SPF WT mice.

While the amount of astrocytes in the hippocampus was not affected by genotype and gut microbiota (Figure 2(g,h)), quantitative morphometric 3D analysis of GFAP^+^ astrocytes showed that in WT mice the lack of gut microbiota results in astrocytes characterized by significantly longer processes and volume relative to astrocytes from SPF housed WT mice (Figure 2(i,j)). In *App*^*NL-G-F*^ AD mice these gut microbiome effects are not seen. Also, by comparing SPF WT with SPF *App*^*NL-G-F*^ AD mice no difference on the level of morphology could be observed. In contrast, GF *App*^*NL-G-F*^ AD mice showed a more amoeba-like astrocyte phenotype compared to GF WT mice.

These data collectively show that in the absence of gut microbiota, the number and ramification of microglia is increased in both WT and *App*^*NL-G-F*^ AD mice, while the phagocytic activity is only decreased in *App*^*NL-G-F*^ AD mice. For astrocytes, only the morphology is affected in WT mice, showing a more ramified phenotype due to the absence of the gut microbiota.

### The absence of gut microbiota reduces microglia-mediated synaptic pruning in App^NL-G-F^ AD mice

**Figure 3.**
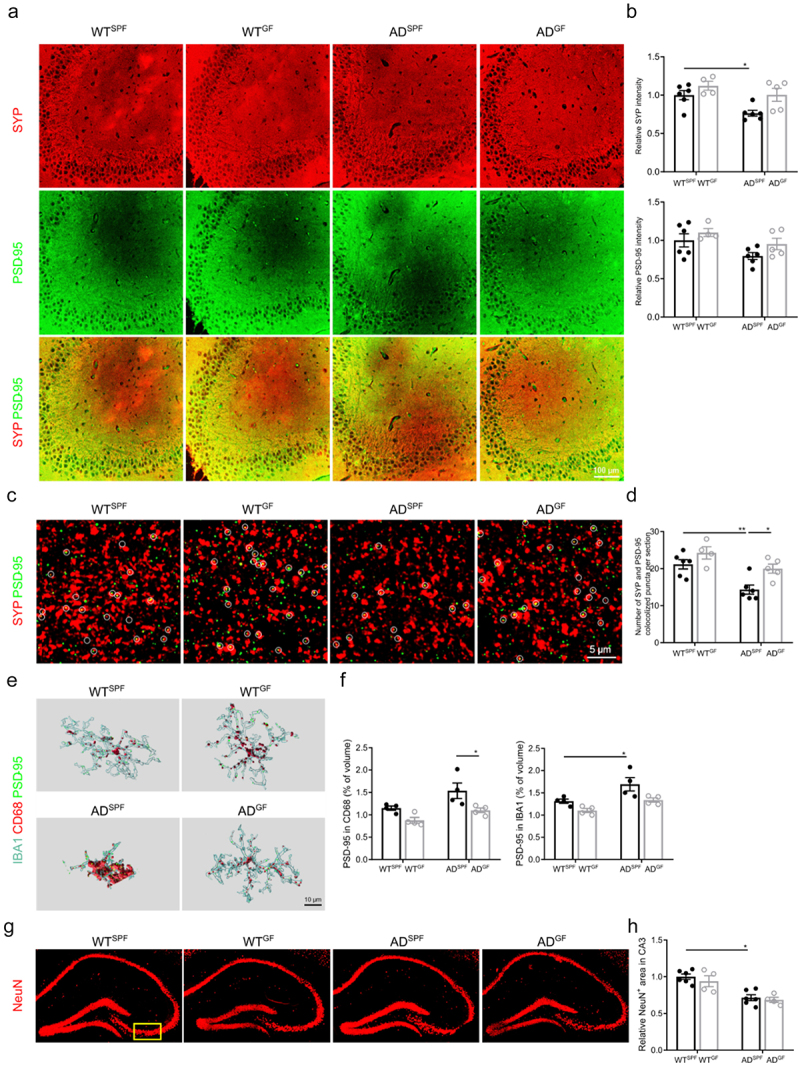
Germ-free housing reduces microglia-mediated synaptic pruning in *App*^*NL-G-F*^ AD mice. (a–b) Representative images (a) and quantification (b) of SYP and PSD-95 co-immunostaining in hippocampus. (c–d) Representative high magnification confocal images (c) and quantification of SYP and PSD-95 co-immunostaining in hippocampus. (e–f) Imaris-based 3D reconstruction (e) and quantification of IBA1/CD68/PSD-95 staining in hippocampus. (g–h) Representative images (g) and quantification (H) of NeuN immunostaining in hippocampus. The graphs are shown as the mean ± SEM and the datapoints are biological replicates (*n* = 4–6). Statistical significance was determined by Kruskal–Wallis test followed by Dunn’s post hoc test. *p < 0.05, **p < 0.01. SPF: specific pathogen-free; GF: germ-free; AD: *App*^*NL-G-F*^ mouse model of Alzheimer’s disease.

Immunostaining of presynaptic protein synaptophysin (SYP) and postsynaptic density protein 95 (PSD-95) showed no significant differences in intensity in the hippocampus between GF and SPF housing of either WT or *App*^*NL-G-F*^ AD mice (Figure 3(a,b)). In line with our previous findings,^6^ SPF *App*^*NL-G-F*^ AD mice show a decrease in SYP intensity compared to SPF WT mice.

Co-localization analysis of both synaptic proteins revealed an increase in double-positive synaptic puncta in *App*^*NL-G-F*^ AD mice under GF conditions compared to SPF housing; WT mice showed a similar trend (Figure 3(c,d)). In line with our previous findings,^6^ SPF *App*^*NL-G-F*^ AD mice show a decrease in SYP-PSD colocalization compared to SPF WT mice.

Moreover, we found that the volume of PSD-95-positive puncta within CD68^+^ phagosomes and IBA1^+^ microglia is decreased in GF housed *App*^*NL-G-F*^ AD mice compared to SPF housed *App*^*NL-G-F*^ AD mice, indicating an increased phagocytosis of synapses by microglia (Figure 3(e,f)).

In contrast, no significant differences in NeuN^+^ area between SPF and GF conditions of *App*^*NL-G-F*^ AD mice was observed (Figure 2(g,h)). Also, here in line with our previous findings,^6^ SPF *App*^*NL-G-F*^ AD mice show a decrease in NeuN^+^ area compared to SPF WT mice.

Combined, these results demonstrate that in *App*^*NL-G-F*^ AD mice the absence of the gut microbiota is associated with a decrease in synaptic deficits and that reduced microglial phagocytosis may contribute to this process. These gut microbiota-dependent effects are not observed in WT mice.

### Isolation and characterization of fecal bEVs

**Figure 4.**
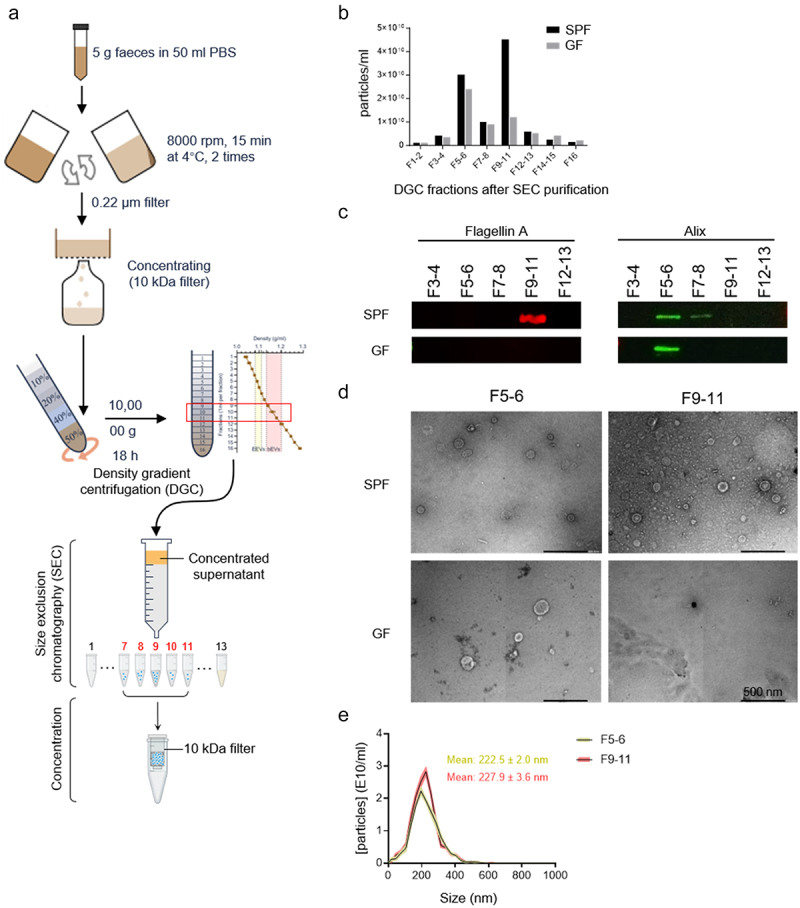
Isolation and characterization of bEVs derived from SPF and GF fecal samples of WT mice. (a) Graphical illustration of fecal bEVs isolation and purification. (b) Particle concentration in different DGC fractions after SEC purification were determined using nanoparticle tracking analysis. (c) Western blot analysis of Flagellin A and Alix in different DGC fractions after SEC purification (10 μg total protein was loaded). (d) Representative negative staining TEM images of DGC F #5–6 and #9–11 after SEC purification. Scale bars: 500 nm. (e) Size distributions of DGC F #5–6 and #9–11 after SEC purification analyzed by NTA (*n* = 3). DGC: density gradient centrifugation; SEC: size exclusion chromatography; SPF: specific pathogen-free; GF: germ-free F: fraction.

To determine the effects of gut microbiota-derived bEVs on brain functions and AD pathology, fecal bEVs were purified from mice housed under SPF and GF conditions by a combination of density gradient centrifugation (DGC) and size exclusion chromatography (SEC) as described previously^28^ (Figure 4(a)). After SEC isolation, the particle number was determined by Nanoparticle Tracking Analysis (NTA), and fraction (F) 5–6 and F9–11 showed high particle numbers for SPF housed samples, while GF-derived fecal samples only showed a peak at F5–6 (Figure 4(b)). The different DGC fractions after SEC purification were checked for enrichment of flagellin A, a major component of flagellar filaments that is also present in bEVs,^32^ and of Alix, an abundant protein in eukaryotic EVs (eEVs).^33^ This revealed that F9–11 contain the bEVs, while F5–8 contain the eEVs (Figure 4(c)). The resulting fractions F5–6 and F9–11 were physically intact when observed under TEM using negative staining (Figure 4(d)), with average diameters of 222.5 ± 2.0 nm and 227.9 ± 3.6 nm, respectively, as determined by NTA (Figure 4(e)). As expected, no bEVs were detected in F9–11 from GF housed fecal samples (Figure 4(d)).

### Commensal gut microbiota-derived bEVs play a critical role in Aβ pathology and microglial activation of App^NL-G-F^ AD mice

**Figure 5.**
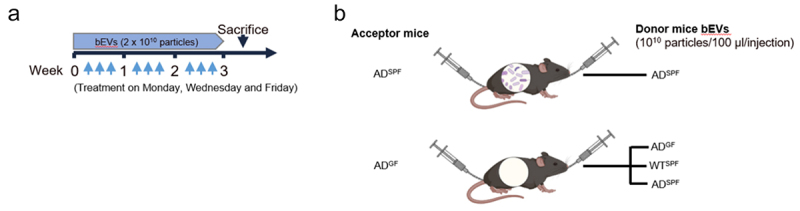
Treatment scheme. (a) Mice were treated with bEVs isolated from fecal slurry through intrarectal and oral administration, receiving equal volumes (2 × 10^10^ particles per injection) simultaneously. If the donor mice were GF housed, an equivalent volume of sample was administered. This regimen was repeated three times per week for three consecutive weeks. (b) Two control groups were included: SPF housed *App*^*NL-G-F*^ AD mice receiving bEVs isolated from the fecal slurry of SPF housed *App*^*NL-G-F*^ AD mice and GF housed *App*^*NL-G-F*^ AD mice receiving bEVs isolated from fecal slurry of GF housed *App*^*NL-G-F*^ AD mice. In addition, GF housed *App*^*NL-G-F*^ AD mice also received bEVs from both WT and *App*^*NL-G-F*^ *AD* SPF housed mice. SPF: specific pathogen-free; GF: germ-free; bEV: bacterial extracellular vesicles; AD: *App*^*NL-G-F*^ mouse model of Alzheimer’s disease.

To examine whether fecal bEVs play a role in the observed differences in AD pathology between SPF and GF mice, fecal bEVs were administered to *App*^*NL-G-F*^ AD mice. Thereto, we administered 2 × 10^10^ particles fecal bEVs both intrarectally and orally, three times a week for three consecutive weeks (Figure 5(a)).

**Figure 6.**
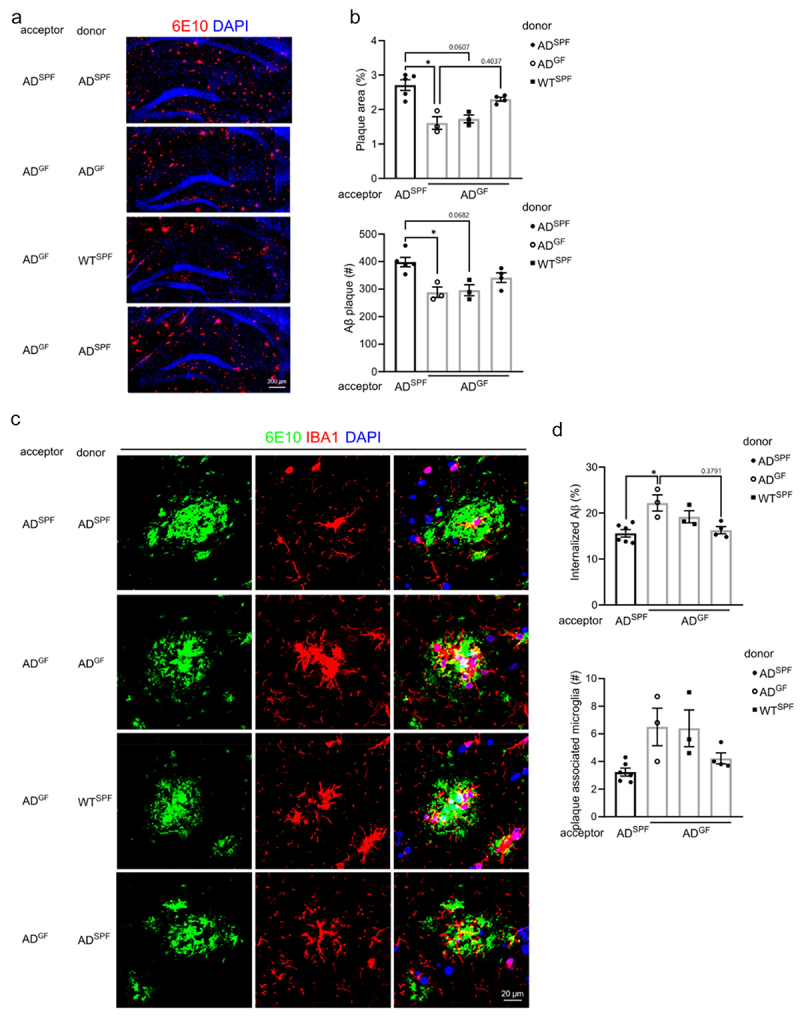
Administration of fecal bEVs affect Aβ plaque load in germ-free *App*^*NL-G-F*^ *AD* mice. Mice were subjected to the experimental setup shown in Figure 5(a–b) Representative images (a) and quantification (b) of 6E10 staining in the hippocampus. (c–d) Representative images (c) and quantification of IBA1^+^ microglia and 6E10 staining in the hippocampus. 3–5 plaques (> 600 μm^2^) are analyzed per mouse. The graphs are shown as the mean ± SEM and the datapoints are biological replicates (*n* = 3–6). Statistical significance was determined by Kruskal–Wallis test followed by Dunn’s post hoc test.*p < 0.05. SPF: specific pathogen-free; GF: germ-free; AD: *App*^*NL-G-F*^ mouse model of Alzheimer’s disease; BEV: bacterial extracellular vesicles.

**Figure 7.**
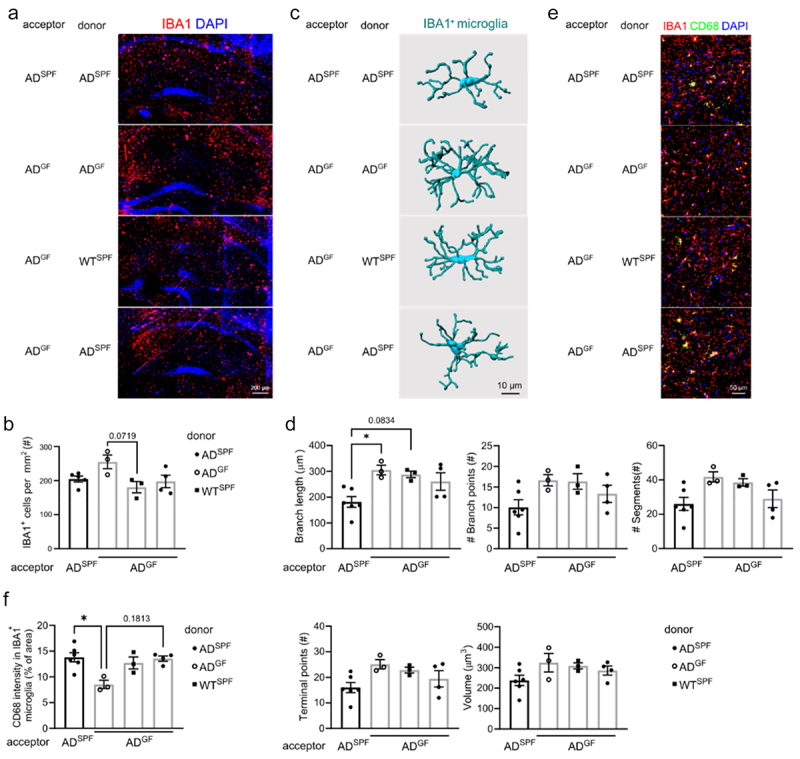
Administration of fecal bEVs to germ-free *App*^*NL-G-F*^ *AD* mice affects microglial phagocytotic capacity. Mice were subjected to the experimental setup shown in Figure 5(a–b) Representative images (a) and quantification (b) of IBA1 staining in hippocampus. (c–-d) Imaris-based 3D morphometric reconstruction analysis (c) and quantification (d) of IBA1^+^ microglia in hippocampus. (e–f) Representative images (e) and quantification (f) of IBA1 and CD68 staining in hippocampus. The graphs are shown as the mean ± SEM and the datapoints are biological replicates (*n* = 3–6). Statistical significance was determined by Kruskal–Wallis test followed by Dunn’s post hoc test. *p < 0.05, **p < 0.01. SPF: specific pathogen-free; GF: germ-free; AD: *App*^*NL-G-F*^ mouse model of Alzheimer’s disease; bEV: bacterial extracellular vesicles.

GF housed *App*^*NL-G-F*^ AD mice were administrated with bEVs from different sources, namely from fecal slurry of SPF housed WT and *App*^*NL-G-F*^ AD mice. To control for the procedure, GF housed *App*^*NL-G-F*^ AD mice received bEVs isolated from the fecal slurry of GF housed *App*^*NL-G-F*^ AD mice and SPF housed *App*^*NL-G-F*^ AD mice received bEVs isolated from the fecal slurry of SPF housed *App*^*NL-G-F*^ AD mice (Figure 5(b)). Next, 3 days after the last administration, mice were sacrificed followed by analysis of Aβ pathology (Figure 6) and microglial activation (Figure 7).

When looking at the effect of commensal gut microbiota derived EVs on Aβ pathology, the control groups showed that the bEV administration had no impact on the previously observed effect: GF versus SPF housed *App*^*NL-G-F*^ AD mice again displayed reduced Aβ pathology as shown in Figure 1. Interestingly, in GF housed *App*^*NL-G-F*^ AD mice, we observed an increase in Aβ plaque area in the hippocampus after administration of fecal bEVs from SPF housed *App*^*NL-G-F*^ AD mice. This increase was not observed with fecal bEVs from SPF housed WT mice (Figure 6(a,b)). Further investigation with IBA1 and 6E10 co-immunostaining shows that the amount of internalized Aβ by hippocampal microglia was decreased in GF housed *App*^*NL-G-F*^ AD mice treated with SPF housed *App*^*NL-G-F*^ bEVs (Figure 6(c,d)). In addition, the number of large Aβ plaque-associated microglia in the hippocampus of GF housed *App*^*NL-G-F*^ AD mice showed a trend toward reduction upon administration of SPF housed *App*^*NL-G-F*^ bEVs (Figure 6(c,d)).

Altogether, these results indicate that bEVs are an important player in the mechanism behind the gut microbiota-induced effects on Aβ pathology in *App*^*NL-G-F*^ mice.

Next, we analyzed the effect of commensal gut microbiota derived EVs on microglia. As expected from Figure 2, the control groups showed that the bEV administration had no impact on the previously observed effect: GF versus SPF housed *App*^*NL-G-F*^ AD mice again displayed increased number of IBA1^+^ microglial cells (Figure 7(a,b)) and a more ramified morphology (Figure 7(c,d)). Administration of bEVs derived from SPF housed WT or *App*^*NL-G-F*^ AD mice to GF housed *App*^*NL-G-F*^ AD mice resulted in limited effects, namely a non-significant decrease in number and ramification of IBA1^+^ microglial cells compared to GF housed *App*^*NL-G-F*^ AD mice that received bEVs from GF housed *App*^*NL-G-F*^ AD mice (Figure 7(a–d)). In addition, the effect of commensal gut microbiota derived EVs on microglia of WT mice was assessed. Similar but non-significant trends were observed in WT mice when looking at the number of IBA1^+^ cells (Figure S1(a – b)).

The fecal bEV effects on microglia was further investigated by IBA1 and CD68 co-immunostaining, showing that higher CD68 immunoreactivity was detected in GF housed *App*^*NL-G-F*^ AD mice administered with SPF housed WT or *App*^*NL-G-F*^ bEVs- compared to the control treated GF housed *App*^*NL-G-F*^ AD mice (Figure 7(e,f)). Similar trends were observed in WT mice, but this was not significant (Figure S1(c – d)).

In addition, as expected from Figure 2, no effects of fecal bEVs treatment were observed on total amount of astrocytes in the hippocampus of *App*^*NL-G-F*^ AD (Figure S2(a – b)) and WT mice (Figure S3(a – b)), but also astrocyte activation did not appear to be affected based on quantitative morphometric 3D analysis of GFAP^+^ astrocytes (Figure S2(c – d)).

## Discussion

During the last years, it has become increasingly clear that gut microbiota can modulate brain function and behavior via the microbiota–gut–brain axis, increasing or decreasing the risks of Alzheimer’s disease (AD).^34^ Currently, gut microbiota dysbiosis has been shown to be involved in the progression of AD in different mouse models.^8,11,35^ The gut microbiota composition of AD patients in clinical studies is also significantly different compared with healthy controls,^36–38^ but the cellular and functional mechanisms of the gut–brain interaction in disease progression are not yet understood.

In general, commensal bacteria are a well-known factor in the training and development of major components of the host’s innate and adaptive immune system.^39^ Specifically, the commensal bacteria play an important role in maintaining microglial maturation and function in the CNS, as evidenced by increased microglial numbers, highly branched arborization and altered gene expression patterns.^40^ Moreover, as early as embryonic brain development, microglia features are essentially controlled by gut microbiota in a gender-dependent manner.^41^ Our result showed that gut microbiota boosted Aβ pathology, which may be caused by decreasing the microglial phagocytosis, as colocalization of microglia and Aβ plaques was lower in the SPF condition compared with the GF condition. Similarly, the gut microbiota has also been found to be detrimental to Aβ pathology in AD mouse models based on overexpression of APP (e.g., APP/PS1 and 3 × Tg) compared to the controls under GF housing conditions or antibiotic (ABX) treatment.^8,11,35,42^ In response to various types of environmental and cellular stress, microglia can rapidly turn to an activated state and change their morphology, phagocytosis capacity, and secretion of cytokines.^43^ Under GF condition, we observed a lower density and less activation of microglia in both WT and *App*^*NL-G-F*^ mice, which was reflected in increases in dendrite length, the number of segments, branch points, and terminal points. Notably, ABX-induced gut microbiota depletion did not show the effects on microglia density in the 5 × FAD mouse model.^42^ These results suggest that microglia may differ significantly in response to constitutive (GF condition) and induced (ABX-treatment) microbiota depletion as well as different AD mouse models. In contrast, astrocytes showed a less reactivated state under GF conditions, but only in WT mice, and this was not seen in *App*^*NL-G-F*^ mice. These findings are in contrast with the existing evidence that the absence of gut microbiota causes a significant reduction in astrocyte reactivity in APPPS_1–21_ mice, underscoring the effect of the used mouse model.^44^ In addition to the Aβ phagocytosis, microglia also help to regulate neuronal function by removing dying neurons, pruning nonfunctional synapses, and producing ligands that support neuronal survival.^45^ In our own and previous observations, microglial hyperactivation is associated with neurodegeneration and excessive synaptic pruning.^6,46^ In the current study, we determined that decreased synaptic deficits in GF conditions were at least partially modulated by microglia phagocytosis. The lack of reactive functional microglia in GF mice results in a loss of synaptic pruning function, which may contribute to increased synaptic density.^47^ Of note, no significant effect of lack of gut microbiota in *App*^*NL-G-F*^ mice on neuronal loss was observed. These results suggest that commensal microbiota may be necessary to support neurogenesis.^48^ Furthermore, the abnormal neuronal activity can directly contribute to the Aβ aggregation, as we observed in our previous study,^6^ and aggregation of Aβ into compact plaques in turn affects local neuronal dysregulation, such as disruption of synaptic integration.^49^ In addition to Aβ accumulation, synapse loss is also closely associated with cognitive decline.^50^ However, further studies are required to elucidate whether these effects ultimately lead to cognitive impairment clearly.

It is well established that microbiota-derived molecules such as LPS, peptidoglycan, and short-chain fatty acids directly modulate neuroinflammation and Aβ accumulation.^6,15,51^ Several of these bioactive molecules are present in microbiota-derived bEVs, i.e., nanosized spherical buds of the outer membrane produced by both Gram-negative and Gram-positive bacteria.^52^ bEVs can transport their cargo to proximal and distal cells to exert physiological and pathological effects.^53–55^ Recently, microbial-derived bEVs have also been demonstrated to be involved in several pathologies, such as intestinal inflammation,^56^ mitochondrial dysfunction,^57,58^ systemic bone loss,^59,60^ pulmonary fibrosis,^61^ and cognitive impairment.^25,62^ Indeed, Wei and colleagues demonstrated that gut microbiota-derived bEVs from AD patients increase BBB permeability and promote the activation of astrocytes and microglia, inducing an inflammatory response and tau hyperphosphorylation by activating the GSK-3β pathway and ultimately leading to cognitive impairment in WT mice.^25^ However, these bEVs were isolated from feces by ultracentrifugation, a method that may result in some contaminants of similar density, such as eukaryotic EVs, lipoproteins and protein aggregates.^21^ These contaminants may have impacts on observed biological functions. In addition, the impact of gut microbiota-derived bEVs on Aβ pathology in AD mouse models, especially under GF conditions, remains unknown. In our study, gut microbiota-derived bEVs were further purified by a combination of density gradient centrifugation and size exclusion chromatography after ultracentrifugation separation according to reported methods.^28^ Post-separation bEVs were characterized using biochemical endotoxin assay in combination with proteins analysis, electron microscopy, and nanoparticle tracking analysis to evaluate bEV quality, purity, abundance, and structure. Based on the results, we could obtain highly purified bEVs with at least eukaryotic EVs removed. Under GF conditions, exposure of *App*^*NL-G-F*^ mice to fecal bEVs from AD mice generates similar effects to those of gut microbiota, leading to the deterioration of Aβ pathology and microglia hyperactivation. Unfortunately, fecal bEVs from WT were not sufficient to further attenuate AD pathology in *App*^*NL-G-F*^ mice under GF housing conditions. These results suggest that bEVs play an important role in gut–brain communication and AD fecal bEVs promote the activation of microglia, accelerating Aβ pathology which may further impair the cognitive function. Both short- and long-term studies are valuable for understanding the effects of continuous bEV exposure on AD pathology. The current experimental design was chosen based on previous findings demonstrating that a single administration of specific bEVs was insufficient to elicit measurable effects. To conduct a long-term study, repeated bEV administration would be necessary over an extended timeframe to maintain their presence and potential impact in the brain. This approach poses significant practical challenges, especially in a germ-free environment, as it demands considerable labor, technical expertise, and a large number of donor mice. While long-term studies represent an interesting direction for future research, our current experimental setup allowed us to effectively capture biologically relevant effects within a more manageable experimental framework.

Similar trends, though not statistically significant, were observed in WT mice receiving bEVs compared to the effects seen in AD mice. This suggests that while bEVs may induce responses on a healthy brain, their impact appears more pronounced in the AD environment, where the brain is already more vulnerable. The presence of underlying neuroinflammation and disease-related changes in AD mice likely amplify the effects of bEVs. This increased susceptibility may be due to factors such as a compromised blood–brain barrier, elevated inflammation, or changes in microglial function caused by the diseases. These findings support the notion that the AD brain is particularly sensitive to external influences, underscoring the significance of our study in understanding the interaction between gut-derived factors and neurodegenerative pathology.

A deeper investigation into the mechanisms by which bEVs affect microglial activation and Aβ pathology would yield valuable insights; however, several challenges hinder such investigations. *In vitro* studies are often hard to interpret because the specific bEVs reaching the brain and their concentrations remain unknown, complicating the establishment of a physiologically relevant model. While we previously have shown bEV cargo delivery in the brain,^23^ it remains unclear whether intact bEVs reach and distribute throughout the brain or if their effects are mediated indirectly through secondary signaling processes. This complexity requires consideration of both direct and indirect mechanisms of microglial activation. In addition, *in vitro* exposure of microglia to bEVs will primarily activate TLR4 signaling due to the presence of LPS, obscuring other potential pathways that might be activated. Given these challenges, further studies are needed to elucidate the precise signaling mechanisms through which bEVs influence neuroinflammation and AD pathology.

Although our study sheds further light on the mechanism of action of gut microbiota in influencing brain function and contributing to AD pathology through secretion of bEVs, there is a lack of studies on specific microbial-derived bEVs as previous studies have shown that specific microbes may be involved in the progression of AD pathology.^11,36^ Comparing bEVs from different bacteria may help to understand further the mechanisms by which gut microbes are involved in AD pathogenesis. For example, our previous studies have shown that gastrointestinal bacteria-*H. pylori*-derived bEVs can cross biological barriers and are subsequently taken up by astrocytes, inducing glial cell activation and neuronal dysfunction via Component 3-C3a Receptor signaling, ultimately leading to worsening of Aβ pathology and cognitive decline.^23^ In addition, *Paenalcaligenes hominis* (*P. hominis*) a member of Proteobacteria, was frequently detected in the elderly but not in children and young adults.^63^ *P. hominis* bEVs may penetrate the brain through the blood as well as the vagus nerve and cause cognitive impairments.^24^ The gut microbiota or specific microbe-derived bEVs may be involved in the same pathways as those described above that influence AD pathogenesis, but this needs to be determined by further research. Furthermore, it is worth noting that the complex impact of the gut microbiota on the pathogenesis of AD may not be a consequence of a particular bacterium or metabolite, but rather brought about as a whole.

## Data Availability

The data that support the findings of this study are available from the corresponding author, V.R.E, upon reasonable request.

## Data deposition

There is no data to deposit.
